# Ecotope-Based Entomological Surveillance and Molecular Xenomonitoring of Multidrug Resistant Malaria Parasites in *Anopheles* Vectors

**DOI:** 10.1155/2014/969531

**Published:** 2014-10-01

**Authors:** Prapa Sorosjinda-Nunthawarasilp, Adisak Bhumiratana

**Affiliations:** ^1^Department of Fundamentals of Public Health, Faculty of Public Health, Burapha University, Chonburi 20131, Thailand; ^2^Department of Parasitology and Entomology, Faculty of Public Health, Mahidol University, 420/1 Rajvithi Road, Rajthewee, Bangkok 10400, Thailand

## Abstract

The emergence and spread of multidrug resistant (MDR) malaria caused by *Plasmodium falciparum* or *Plasmodium vivax* have become increasingly important in the Greater Mekong Subregion (GMS). MDR malaria is the heritable and hypermutable property of human malarial parasite populations that can decrease *in vitro* and *in vivo* susceptibility to proven antimalarial drugs as they exhibit dose-dependent drug resistance and delayed parasite clearance time in treated patients. MDR malaria risk situations reflect consequences of the national policy and strategy as this influences the ongoing national-level or subnational-level implementation of malaria control strategies in endemic GMS countries. Based on our experience along with current literature review, the design of ecotope-based entomological surveillance (EES) and molecular xenomonitoring of MDR falciparum and vivax malaria parasites in *Anopheles* vectors is proposed to monitor infection pockets in transmission control areas of forest and forest fringe-related malaria, so as to bridge malaria landscape ecology (ecotope and ecotone) and epidemiology. Malaria ecotope and ecotone are confined to a malaria transmission area geographically associated with the infestation of *Anopheles* vectors and particular environments to which human activities are related. This enables the EES to encompass mosquito collection and identification, salivary gland DNA extraction, *Plasmodium-* and species-specific identification, molecular marker-based PCR detection methods for putative drug resistance genes, and data management. The EES establishes strong evidence of *Anopheles* vectors carrying MDR *P. vivax* in infection pockets epidemiologically linked with other data obtained during which a course of follow-up treatment of the notified *P. vivax* patients receiving the first-line treatment was conducted. For regional and global perspectives, the EES would augment the epidemiological surveillance and monitoring of MDR falciparum and vivax malaria parasites in hotspots or suspected areas established in most endemic GMS countries implementing the National Malaria Control Programs, in addition to what is guided by the World Health Organization.

## 1. Current Malaria Risk Situations

Malaria is a mosquito-borne parasitic disease of humans. This poverty-associated disease accounts for more than 200 million malaria cases annually reported between 2001 and 2012 in 104 endemic countries [1]. Of the 104, 99 endemic countries with ongoing malaria transmission include 79 being in malaria control phase and the same 10 that achieved preelimination and elimination phases. Endemic countries in the malaria control phase employ surveillance, prevention, and control, focusing mainly on* Plasmodium falciparum* causing mortality and morbidity and* Plasmodium vivax* causing chronic morbidity. In Africa alone, life-threatening malaria poses certain risks of mortality and morbidity among vast populations in 45 Sub-Saharan African countries [1]. Outside of Africa, malaria risk situations taking place in the populations at risk are greatly variable. For example, the Greater Mekong Subregion (GMS) countries—comprising Cambodia, Lao People's Democratic Republic, Myanmar, China (Yunnan, China), Thailand, and Vietnam—have been challenging the emergence and spread of multidrug resistant (MDR) falciparum and vivax malaria—as well as artemisinin resistance in* P. falciparum* and chloroquine resistance in* P. vivax*—across the international borders within the GMS countries [2–8]. Other certain malaria risk situations in the Southeast Asia include malaria associated with rubber plantations and on the borders in Thailand [9, 10] and the emergence of* Plasmodium knowlesi* in Malay Peninsula [11–13].

Of note, the MDR malaria is demonstrated by the ability of heritable and hypermutable human malarial parasite populations that can decrease* in vitro* and* in vivo* susceptibility to proven antimalarial drugs as they exhibit dose-dependent drug resistance and delayed parasite clearance time in treated patients. As the result of the selection pressures over time, the multigenic MDR falciparum or vivax parasite is genetically determined, but not yet completely understood, by MDR gene functions. Nevertheless, this MDR malaria is normally not unraveled using the notifiable disease surveillance system that routinely identifies demographic and geographic differentiation and assesses trend in malaria incidence, that is, a number of autochthonous malaria cases or newly infected cases, whose blood sample is diagnosed with known* Plasmodium* infection origin, that regularly occur over a time period. Malaria epidemiology has been so far a basic science of what factors contribute to the infection in individuals and how communicable is malaria in the population at risk over space and time. But we often lack transdisciplinary knowledge of what is the link between changes of malaria landscape ecology and epidemiology in different epidemiological complex settings around the globe [14, 15]. This might influence the ongoing national-level or subnational-level implementation of malaria control strategies and operational response activities [1, 2]. Thus, if continually unbridled, such MDR malaria risk situations in the GMS and Southeast Asia might reflect consequences of both formulating the national policies and strategies and operating strategic deployment in most affected countries. In other words, current MDR malaria offers the challenges to policy makers, public health professionals, health planners, and scientists. Accordingly, strategic approaches to more appropriately designed surveillance and control and practical solutions should be developed with the contexts of each endemic country. Particularly, the itinerant populations including mobile persons and migrant workers must be logically analyzed so as to exploit the adaptations of interventions and services applied to or used in both surveillance and control for the risks of MDR malaria along the international borders between the GMS countries [2], as there exist some vulnerabilities described below.

## 2. Surveillance and Control Attributed to MDR Malaria Risk

With ongoing malaria transmission, the endemic GMS countries implement the National Malaria Control Programs (NMCPs) in the malaria control phase—aiming at reducing the malaria mortality and morbidity rates by interrupting or curtailing transmission and diminishing human-vector contact. The NMCPs—subsidized by the Global Fund Malaria (GFM) Program—employ global malaria control strategies, which include early diagnosis and prompt treatment using rapid diagnostic tests (RDTs) and artemisinin-based combination therapies (ACTs) and other vector control using insecticide-treated nets (ITNs)/long-lasting insecticidal nets (LLINs) in combination with indoor residual spraying (IRS) [2, 9, 10]. To achieve the ultimate goal of the NMCP, several key factors have to be addressed: (i) scaling up coverage and expansion services of these pragmatic control strategies synergistically implemented in all geographically defined transmission control areas (TCAs), (ii) alleviating control activities and measures to be applied to or used in the process of health behavioral changes of local people or at-risk groups, (iii) gathering and leveraging data/information required for monitoring and evaluation of the effectiveness of the implementation of malaria control strategies, (iv) forecasting the malaria outbreaks or epidemics of MDR malaria and analyzing the vulnerability in how the emergence and spread of MDR falciparum and vivax malaria, as well as* Anopheles* vector resistance to insecticides, occur in hotspots or suspected areas, (v) averting and abating the epidemics of MDR falciparum and vivax malaria and insecticide resistance in malaria vectors, and (vi) fund-raising, resourcing either nationally or internationally, and, more importantly, mobilizing the resources not only for strengthening capacity building in diagnosis, surveillance, and control within endemic countries but also for increasing expenditures of universal access to healthcare services and malaria control management activities against malarial parasites and mosquito vectors.

To reduce morbidity and mortality, the GFM-subsidized NMCPs in endemic GMS countries implement early diagnosis and prompt treatment for uncomplicated malaria. This strategic approach is applied to the TCAs where the community-outreach healthcare service units such as malaria mobile clinics, malaria control units, or malaria posts are deployed as the parts of the GFM-supported NMCPs. These community-outreach healthcare facilities—to which the affected people and community can freely access—can provide blood examination and appropriate prompt treatment. Susceptibility to malaria depends on exposing the infections likely due to factors such as settlements, movement activities, occupations, perception and awareness, health behaviors, and access to healthcare services. However, some infected persons may seek blood examination and treatment either at peripheral healthcare services located within the TCAs or at different levels of other public or private healthcare services located outside the TCAs. For instance, certain risk situations of MDR malaria are likely to occur if any infected individuals—residing in remote pocket of endemic village where malaria post is established—can pose the risk for either misdiagnosis with RDTs, treatment delay, or loss to follow-up treatment with ACTs. As such, some vulnerabilities that constrain routine malaria surveillance and control vertically implemented in the TCAs as the parts of the NMCPs [2] can be summarized as follows.

- Some malaria-contracted persons carrying any infection, whether single or mixed, are sometimes diagnosed as negative with a RDT and subsequently lost to follow-up blood examination. During the prodromal period, the increased risks for the spread of MDR malaria are associated with the occupation, movement activity, and lack of preventative action. Consequently, the vulnerability is due to the combination of treatment delay, improper self-medication or healthcare seeking, and prolonged exposure to multiple bites of* Anopheles* vectors at multiple locations.
- When on-site diagnosed as positive with a RDT, any malaria cases are given either a recommended ACT for* P. falciparum* or first-line treatment for* P. vivax* and then followed up. Such MDR malaria risks are likely to increase due to low adherence by the patients, the lack of effective point-of-care monitoring of the infection after treatment, and the insufficient availability of other well-trained paraclinical staff (e.g., public health nurses, community health workers, and infection control personnel). Moreover, paraclinical staff cannot persistently perform foci investigation and follow-up treatment unless surveillance and reporting system are organized and coordinated with the malaria posts.
- In MDR malaria-associated international border settings, any local persons who cross the border between the countries are likely to be frequently exposed to multiple bites of* Anopheles* vectors at multiple locations [2]. Cross-border immune malaria cases carrying the* Plasmodium* infections are asymptomatic during the prodromal infection. The infection may or may not be epidemiologically linked with the time and location at which they came into close contact with infective bite(s) of* Anopheles* vectors. When seeking diagnosis service at any peripheral healthcare facility in any land border settings, they may have delayed blood examination and treatment. That is, the longer the prodromal period of infection, the greater the risk of the spread of MDR malaria.
- The vulnerability will increase if the weakness of the NMCP implementation remains unsolved. There are some problems such as low degree of perception and awareness of local people at risk, inappropriate resource mobilization, and improper healthcare management (i.e., unorganized and less coordinated). These plausible factors might constrain the effective implementation and coverage of early diagnosis and prompt treatment strategy, even though the GFM-supported NMCPs corroborate the existing public health systems and healthcare services that are well organized and sufficient.

From national and regional perspectives, the emergence and spread of MDR falciparum and vivax malaria against ACTs or artemisinin derivatives [2]—as well as* Anopheles* vector resistance to pyrethroid insecticides [16]—in hotspots or suspected areas have increasingly become important as they have the potential to jeopardize the management activities and desired outcomes of the ongoing implementation of the existing NMCPs—whether separately or jointly—in the GMS, Southeast Asia, and South Asia. These agendas have recently been considered national and regional public health concerns in collaboration with the World Health Organization (WHO) and other internationally enterprising counterparts [2–8, 16]. The challenge is that surveillance and monitoring systems used in the existing NMCPs have to be augmented to improve the efficacy of ACTs through which MDR malaria-carrying persons are effectively monitored both timely and reliably to determine the extent to which the parasite is resistant against ACTs, in parallel to examining malaria vectors that carry MDR malaria. For example, the consequences of MDR malaria reflect preparedness and responses to the epidemics of MDR falciparum and vivax malaria geographically prone in most endemic GMS and Southeast Asian countries that implement the GFM-supported NMCPs [9]. Without the development and enhancement of malaria surveillance systems, the routine diagnosis and epidemiological surveillance will no longer be effective enough to detect any anomalous infections in hotspots or suspected areas infested with any* Anopheles* vectors that carry MDR malaria parasites. How can we enhance the capacity of surveillance and monitoring systems as key components of the NMCPs used in detection and identification of unusual situations and trend of MDR malaria?

## 3. Changes in Malaria Landscape Ecology and Epidemiology and Ecotope-Based Entomological Surveillance

To reduce MDR malaria risk situations that possibly occur in the TCAs as mentioned above, the NMCPs require the design of ecotope-based entomological surveillance (EES) and monitoring systems that would be effective and available for use in detection and identification of MDR malaria parasites in hotspots or suspected areas [2, 10]. This framework should be complimentary to the existing epidemiological surveillance as the part of the NMCP. The existing epidemiological surveillance encompasses the ongoing and systematic processes of up-to-date and validated data collection, collation, analysis, and dissemination of information. Response must rely on (i) identifying demographic/geographic differentiation and assessing trend in which malaria cases are notified timely and reliably using routine surveillance and reporting systems, (ii) detecting and identifying the infections with emerging MDR parasites by using molecular marker-based polymerase chain reaction (PCR) detection methods, (iii) assessing the efficacy of ACTs for uncomplicated* P. falciparum* patients or first-line treatment for* P. vivax* patients by using the gold standard* in vitro* drug sensitivity tests, or by using other* in vivo* susceptibility or therapeutic efficacy tests [2], and (iv) analyzing the vulnerability in how vulnerable persons are exposed to the infections as well as what factors influence their seeking diagnosis and treatment, adherence of follow-up diagnosis and/or treatment, and compliance with a recommended ACT for* P. falciparum* or first-line treatment for* P. vivax* [2].

In parallel, the EES must leverage needed data/information to (i) determine the magnitude and geographical distribution to which* Anopheles* vector resistance to insecticides is related and (ii) analyze the vulnerability of* Anopheles* vectors that are adapted to local environments—whether or not ecological changes occur—in transmission areas geographically associated with or prone to MDR malaria. More significantly,* Anopheles* vectors—carrying the infections with MDR malaria parasites—must be monitored timely and reliably in the TCAs. Subsequently, the hotspots or suspected areas of the spread of MDR falciparum and vivax malaria can be logically analyzed. The existing NMCPs normally target the coverage of implementing malaria control strategies in the TCAs rather than extending services to transmission-prone areas. However, this is the challenge in which (i) the NMCP coordinators should pay particular attention to MDR malaria risks associated with changes of landscapes (i.e., ecotopes and ecotones) in which local people are at risk of exposure to infective bite(s) of local* Anopheles* vectors (whether primary or secondary) and (ii) they should demarcate the most likely high risk areas (i.e., hotspots and suspected areas of MDR malaria). Thus, if landscape ecology changes occur in the TCAs, we should understand how significant the malaria ecotope and ecotone are as described below.

### 3.1. Malaria Ecotope and Ecotone

**Figure 1:**
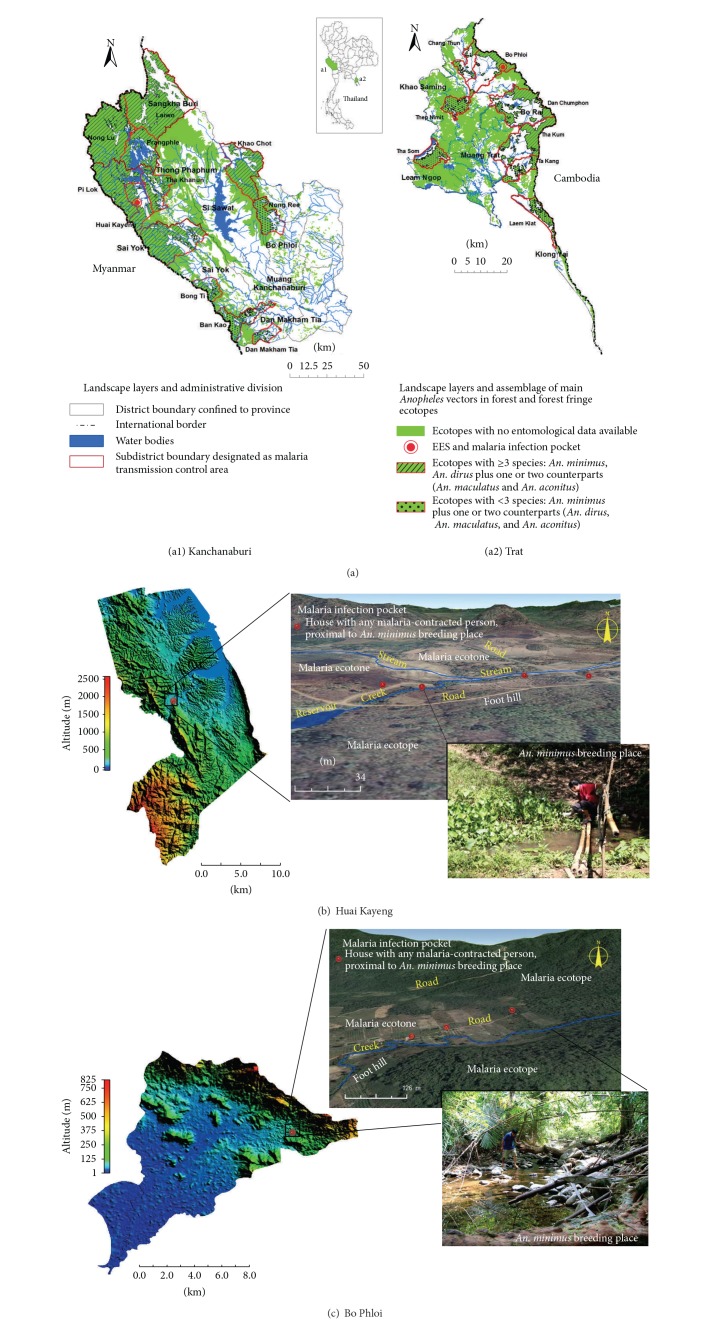
Maps of forest and forest fringe ecotopes of malaria endemic provinces of Thailand. ((a1)-(a2)) Forest and forest fringe ecotopes of malaria endemic provinces, Kanchanaburi and Trat, accommodate the assemblage of main* Anopheles* vectors. Mapping-based areas (km^2^) of forests and forest fringes and water bodies are 7,247.97 and 622.87 for Kanchanaburi and 943.63 and 6.61 for Trat. Some subdistrict-level administrative areas of the provinces are shown for malaria transmission control; all of which establish diverse malaria ecotopes and ecotones through changes of land use and land cover patterns pertaining to human activities. Only the subdistrict used in the EES and for establishment of the malaria infection pocket is representative of each province. ((b)-(c)) In Huai Kayeng and Bo Phloi subdistricts, two different malaria infection pockets confined to the TCAs are established for the EES. During September-October 2011, the susceptible persons who developed indigenous malaria were all thought to acquire naturally the infection through bite(s) of potent* Anopheles* vectors that breed and/or forage close to the patients' houses. Main drivers are human settlements and activities pertaining to agricultural intensification of the rubber plantations. Among the responsible anophelines,* An. aconitus* and* An. dirus* were found to carry MDR vivax malaria parasites during which a course of follow-up treatment of the notified* P. vivax* patients who received the first-line treatment was done. All topographic maps were performed using the ArcGis ver. 10.0 for the landscape layers of data sources (forest patches, water bodies,* Anopheles* vector assemblages, and administrative divisions), Global mapper ver. 11.0 for the elevation data, and the Google Earth for the topography.

The EES relies on the coexistence of any currently or newly malaria-developing cases and a diverse group of* Anopheles* vectors—as both endogenous and exogenous counterparts can assemble in any given malaria ecotope. This means that a plethora of* Anopheles* mosquitoes, including malaria vectors, can infest both malaria ecotope and malaria ecotone (Figure 1). Currently, Thailand is experiencing malaria landscape ecology and epidemiology changes [2, 9, 10] and perhaps the similar phenomena occur in malaria endemic settings in the GMS and Southeast Asia. A malaria ecotope is a malaria transmission area geographically associated with the infestation of* Anopheles* vectors and particular environments to which the interconnections of humans and environments favorable to breeding and feeding are related. A malaria ecotone is a transition zone between two different local environments in a given malaria ecotope or between the malaria ecotopes. Geographically defined malaria ecotope, on the other hand, encompasses the topological landscapes (land use and land cover patterns) that are composed of a number of landscape classes (Figures 1(a)-(a1) and (a2)) [10]. Each class can be demarcated as a closed plane figure (generally polygonal) and slope of varying angles of inclination and elevation. Thus, malaria ecotope creates microclimatic environmental conditions contributing to ecosystem diversity of malaria vectors and their symbiotic counterparts as the result of the biological, physiological, and chemical pathways and processes that promote adaptation, survival, and diversification.

In given malaria ecotope, the adaptation (abundance and distribution) and survival (growth and development) of the anopheline mosquito populations are physiologically regulated by climatic conditions [17–23]. Also, the regulation of the anophelines is influenced by species-species interactions, predator-prey interactions, and aggregating-segregating forages [24–29]. The diversification of* Anopheles* vectors—including their sibling species and counterparts—can be measured by richness of species and populations or by genetic diversity in the populations and communities [19, 21, 23, 29–38]. In Thailand, there have been lines of evidence that three main malaria vectors play roles in malaria transmission in the forest and forest fringe ecotopes. The primary vectors include* An. dirus*,* An. minimus*, and* An. maculatus*. The secondary vectors include* An. aconitus*,* An. pseudowillmori*, and* An. sundaicus*. The suspected vectors include* An. barbirostris*,* An. campestris*,* An. philippinensis*, and* An. culicifacies*; all of which are known to be potent malaria vectors in other endemic countries of South and Southeast Asia. Nonetheless, the only 5* Anopheles* species complexes (i.e.,* An. dirus* complex,* An. minimus* complex,* An. maculatus* complex,* An. sundaicus* complex, and* An. barbirostris* complex) are among the responsible malaria vectors that have been well established for their adaptation to local environments in the forest and forest fringe ecotopes in Thailand, based on the abundance, geographical distribution, behaviors, and genetic diversity of the sibling species members pertaining to the epidemiologic implications [23, 39, 40]. Dirus Complex has 5 sibling taxa (e.g.,* An. dirus*,* An. baimaii*,* An. nemophilous*,* An. cracens*, and* An. scanloni*). All of which are indigenous to the forest ecotopes but* An. dirus* is the predominant species (Figure 1). Only two sibling taxa,* An. dirus* and* An. baimaii*, are adapted to local environments relating to the forest and forest fringe ecotopes but they are intolerant to environmental changes as the result of agricultural intensifications (i.e., the production activities of monocultural agroforestry, crop plantations, or other livestock in farmland). Minimus Complex has only 2 sibling taxa (e.g.,* An. minimus* and* An. harrisoni*), which are endogenous to the forest and forest fringe ecotopes.* An. minimus* is the predominant species that distributes widely across Thailand (Figure 1) and is adapted well to local environments with agricultural practices and irrigation or environmental changes as the result of agricultural intensifications. Maculatus Complex has 7 sibling taxa (e.g.,* An. maculatus*,* An. sawadwongporni*,* An. pseudowillmori*,* An. willmori*,* An. dravidicus*,* An. notananai*, and* An. rampae*); all of which are endogenous to the forest and forest fringe ecotopes. Two sibling taxa,* An. maculatus* and* An. pseudowillmori*, are the predominant species that can adapt well to local environments pertaining to agricultural practices and irrigation across Thailand (Figure 1).* An. maculatus* is the primary malaria vector, as its sibling species counterparts,* An. pseudowillmori* and* An. sawadwongporni*, are considered the secondary and suspected vectors. Sundaicus Complex has 5 sibling taxa (e.g.,* An. epiroticus* (*An. sundaicus* A),* An. sundaicus *B,* An. sundaicus* C,* An. sundaicus *D, and* An. sundaicus *E), but only* An. epiroticus* is autochthonous to the forest and forest fringe ecotopes constituted of coastal or island ecosystem in the East and South of Thailand. Barbirostris Complex has also 5 sibling taxa (e.g.,* An. barbirostris* A1,* An. barbirostris* A2,* An. barbirostris* A3,* An. barbirostris* A4, and* An. campestris*); all of which distribute widely across Thailand. Four sibling taxa,* An. barbirostris* A1 to A4, are autochthonous to the forest and forest fringe ecotopes, whereas* An. campestris* is adapted well to low-lying areas with agricultural practices and irrigation as well as urban and built-up land. Together, the population biology of the adapted* Anopheles* vectors remains to be established, especially in such changes in malaria landscape ecology and epidemiology.

Accordingly, some potent* Anopheles* faunas—including* An. dirus*,* An. minimus*, and* An. maculatus*—play important role in seasonal transmission of forest and forest fringe-related malaria that usually affects local people with agricultural practices in Thailand, GMS, and Southeast Asia. These* Anopheles* vectors promote vertical transmission to humans due to their feeding habit, human host preference, and abundance/distribution by seasonal variation [18, 20–26, 28, 29]. If the occupational and behavioral exposures to exophagy (outdoor biting) occur, the susceptible persons will have increased risk for malaria and hence they will pose the risk for prevention and control. Increased risks are also accelerated by certain combinations of human settlements, movement activities, and agricultural intensifications in the forests and forest fringe areas. This is a reason why the occupational and behavioral exposures render the adults susceptible to occasionally acquire the infection in infection pockets in the responsible malaria ecotopes (Figures 1(b) and 1(c)) [2, 9, 10]. Moreover, human settlements and movement activities—to which the particular environment of human-vector contact is related—are likely to contribute to demographical and geographical differences in malaria incidence. Thus, MDR risk is more likely to be dynamic as there is a larger increase of agricultural intensification (changes in land use/land cover patterns), resulting in greater diversity in malaria ecotopes and ecotones (Figures 1(a)-(a1) and (a2)) in the TCAs.

A pocket of the infection is a transmission focus in which any susceptible person acquires naturally the infection through the bite(s) of primary or secondary* Anopheles* vector(s). For example, Figure 1 shows two different malaria infection pockets confined to the TCAs of Huai Kayeng (Figure 1(b)) and Bo Phloi (Figure 1(c)). Malaria transmission foci relate local environments to favor either breeding or foraging of* Anopheles* vectors that can transmit malaria parasites vertically to humans and,* vice versa*, the vectors become infected whenever taking gametocyte-carrying blood meal from any malaria carrier. Thus, the infection pocket is considered the smallest assessment unit, which is confined to a given malaria ecotope. Any infection pocket in a malaria ecotope corresponds to a unique landscape class or overlapping landscape classes in which human-vector contact can occur over time. A malaria ecotope may have one or more infection pockets, depending primarily on the susceptibility of vulnerable persons or groups that vary human settlements, movement activities, occupations, and behaviors. If the human settlement is confined to the same infection pocket (Figures 1(b) and 1(c)), any indigenous persons at risk will naturally acquire one* Plasmodium* infection or mixed infection through biting of* Anopheles* vectors whether indoors or outdoors due likely to their nonpreventable actions during the night time [9, 10]. If the human settlement is located apart from the infection pocket, any exposed individuals at risk may be challenged to acquire the infective bite in nearby pockets, likely due to the combination of the movement or workmen's forest activity and lack of preventive behaviors during the night time [2, 9, 10]. Thus, either* Anopheles* vectors carrying the infection or vulnerable individuals acquiring the infection over time may not always have the same source of infection.

#### 3.1.1. Ecotopes of Malaria-Associated Forests and Forest Fringes

**Figure 2:**
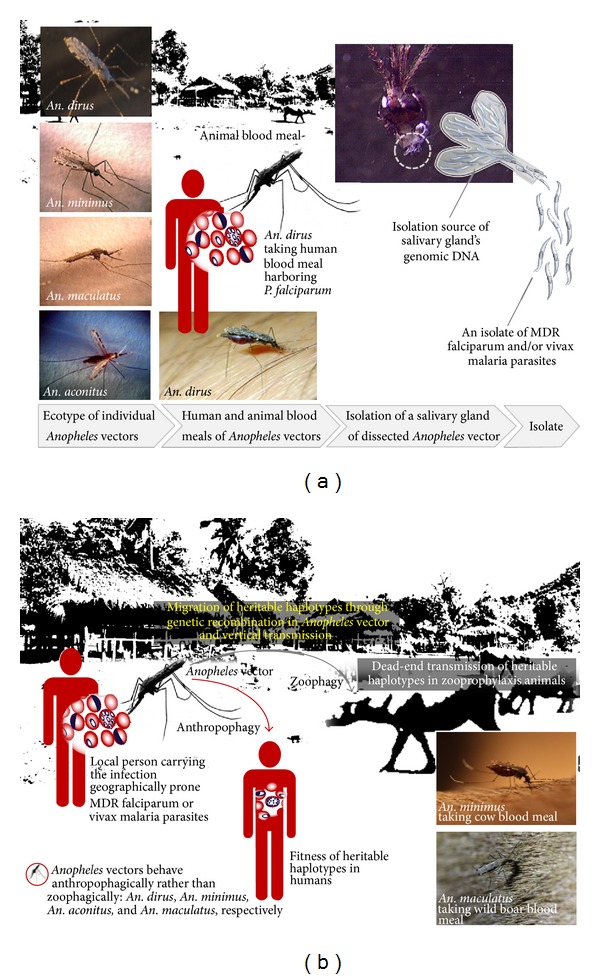
*Anopheles* vectors and MDR malaria haplotypes. (a) A framework for the EES which can permit the downstream procedures for both the identification of the wild-caught pools or individuals of* Anopheles* vectors and the detection and identification of MDR malaria parasite isolates present in salivary gland DNA of individual* Anopheles* vector. Such malaria ecotopes of forest/forest fringes shown in Figure 1 can provide isolation sources of four main* Anopheles* vectors (e.g.,* An. dirus*,* An. minimus*,* An. maculatus*, and* An. aconitus*). (b) A framework for the molecular xenomonitoring of MDR malaria which can permit the analysis of anthropophagous* Anopheles* vectors carrying MDR malaria parasites present in any malaria infection pocket of the forest/forest fringe ecotope. Based on molecular markers for putative drug resistance, any haplotypes of MDR malaria parasites originally obtained from the* Anopheles* SG DNA are hypothetically advantageous parasite population under the selection pressures over space and time.

As mentioned earlier, bionomics of the* Anopheles* vectors that can infest or reinfest different endemic settings is requisite for the EES and molecular xenomonitoring of MDR malaria parasites. In Thailand, typical malaria ecotopes—contributing to MDR risks—are geographically associated with the forests and forest fringes (e.g., highland areas of rubber plantations and/or mixed orchards) to which* Anopheles* vectors are sessile or adapted [10]. Ecotopes of coastal and island malaria do not contribute greatly to MDR malaria risks. These responsible malaria ecotopes are likely to create microenvironments favorable to breeding and/or feeding of potent* Anopheles* vectors (e.g.,* Anopheles dirus*,* An. minimus*,* An. maculatus*, and* An. aconitus*) (Figures 1 and 2(a)) [10]; all of which are epidemiologically important for surveillance, prevention, and control.

In most TCAs of Thailand and its international borders,* An. minimus* rather than* An. dirus* [10, 18, 19, 23, 28, 36–38] is adapted well to the ecotopes of forests and forest fringes such as rubber plantations (Figure 1). These ecotopes have diverse landscape classes that can promote reproduction and foraging of the anophelines. Landscape classes include evergreen forest (whether hilly, perennial, moist, dry, or mixed) and monoculture plantations (e.g., rubber and/or mixed orchards), with irrigation and other water bodies (i.e., slow running creeks rather than streams). The abundance and distribution of* An. minimus* are seen all year-round but there is fluctuation in number and density; peak density varies between rainy season (June-July) and winter season (January-February) [10, 17, 18, 23–25, 28]. Soon after declining rainfalls until the early winter,* An. minimus* and 3 other potent counterparts become aggregated during the seeking of blood meal in the ecotopes of forest fringes and rubber plantations [10, 17, 41]. Of these,* An. aconitus* and/or* An. maculatus* is relatively abundant in some rubber plantations in the South and the East of Thailand. During winter season,* An. aconitus* is still dominant as compared to its counterparts,* An. maculatus*,* An. minimus*, and* An. dirus*, respectively.* An. minimus* and its counterparts (*An. aconitus* and* An. maculatus*) behave both anthropophagically and zoophagically whereas* An. dirus* has a preference of feeding blood meal on humans rather than on animals [10, 18, 22, 23, 26–29, 41]. In some cases,* An. dirus* prefers feeding animals rather than humans. If there is the existence of livestock in any farmland in the relevant malaria ecotopes, these 3 potent vectors prefer attacking animals during seeking any blood meal outdoors (Figure 2(b)). Together, the infection pockets proximal to malaria patients' houses are epidemiologically linked with the other data of breeding site, foraging, feeding habit, season regulation, and topography. These following parameters are considered essential to determine* Anopheles* sampling sites suited to the EES and molecular xenomonitoring of MDR malaria parasites in the* Anopheles* vectors.

#### 3.1.2. Ecotopes of Malaria-Associated International Borders

The international borders within the GMS promote more greatly variable malaria ecotopes because there exist complex epidemiological settings due to intense movement of cross-border migrant workers and border crossing of local people residing on or close to the borders [2, 10, 36, 37, 42, 43]. In border malaria, cross-border local people who are normally immune may often revisit the forests alongside the border. The susceptible persons occasionally acquire the MDR malaria infections as they are epidemiologically linked with frequent exposure to multiple bites of* Anopheles* vectors at multiple locations [2, 10]. Thus, epidemiological surveillance and foci investigation may not always relate the infections or the spread of MDR malaria parasites to any hotspots or suspected areas confined to the TCAs in any land border settings [2].

International borders offer the challenges, especially in establishing the procedures for and the activities of the EES as well as epidemiological surveillance and monitoring of MDR malaria because it is difficult to identify any infection pocket confined to a malaria ecotope. However, detection and identification of* Anopheles* vectors responsible for the emergence and spread of MDR malaria can be done logically and expediently—following human-vector contact and recall of the exposure situation based on time which passed before the symptoms become evident. For example, the malaria ecotope available for the EES for MDR malaria is logically analyzed when any suspected malaria cases who seek diagnosis services at a malaria post are examined as positive by a RDT and followed by a recommended ACT for* P. falciparum* or first-line treatment for* P. vivax* [2, 44]. By using the previously mentioned principal parameters, certain or suspected pockets can be deduced and targeted for the foci investigation and the EES.

### 3.2. *Anopheles* Ecotype and MDR Malaria Haplotype

A malaria ecotope creates a plethora of* Anopheles* mosquitoes including the primary and secondary vectors, which can adapt and survive in their fitted local environment(s). Assuming that ecological changes can alter the adaptation and survival of the* Anopheles* vectors in various malaria ecotopes, we need to collect and leverage entomological data/information on the ecotypes of local* Anopheles* vectors—adapting to their local environments. The raised questions ask how the local* Anopheles* vectors play a role in the spread of MDR falciparum or vivax malaria parasites as well as how they provoke the insecticide resistance. Here we address only the significant role of the* Anopheles* ecotypes that generate a diversification of MDR malaria haplotypes (Figure 2(b)).

The ecotype of* Anopheles* vector is a genetically unique population in a given* Anopheles* species that is adapted to its local environment in a geographically defined malaria ecotope, as the individuals exhibit morphologic, physiologic, and behavioral characteristics distinguishable from other populations of* Anopheles* species (Figure 2(a)).* Anopheles* vectors, of which the ecotypes are sessile to such a particular climatic environmental condition in such malaria ecotope, may have the adaptation to an ecotone, in which the population dynamics of different ecotypes of* Anopheles* vectors and their counterparts possibly occur (Figures 1(a)-(a1) and (a2)). Such adapted ecotype of* Anopheles* vector can exploit host-seeking strategy either anthropophagically or zoophagically in malaria ecotope or ecotone. Therefore, malaria transmission influenced by the principal* Anopheles* vector in specific malaria ecotope may not spread directly to other different malaria ecotopes because there is geographic difference in their ecotone which regulates the adaptation and survival of the ecotypes of those responsible* Anopheles* vectors.

A theorem of MDR malaria transmission—naturally occurring in specific malaria ecotope—can be explained by a theoretical number of the haplotypes of MDR malaria parasites, based on the selection of the genetically associated MDR genes. The geographically associated MDR haplotype can be referred to as a set of inheritable alleles that escape under the pressures of the selection on genetically linked MDR genes. Diversity in the structure of the gene pool of MDR malaria parasite population is a trade-off. Hence, the cost of fitness that exists in geographically defined malaria ecotope is a consequence of reproduction in specific hosts. A haplotype is the mixture of the genotypes of a polymorphic gene, as it is not easily identified because a mixture of those of the two or more alleles does exist in the diploid malaria parasite organism as the result of modality of recombination in* Anopheles* vector (Figure 2(b)). That is, a MDR haplotype is genetically determined by a known set of polymorphic drug resistance markers; the details are described later. If MDR malaria parasites interbreed in the population via bites of* Anopheles* vector(s) in certain local environment(s) in malaria ecotope,* P. falciparum* or* P. vivax* isolates originally obtained from infected humans or* Anopheles* vectors will be likely to create haplotype homozygosity and, eventually, to reduce genetic variations of the* P. falciparum* or* P. vivax* MDR parasite populations. The homozygosity of haplotype (genotypes) of the putative drug resistance genes spanning on the homologous chromosome of* P. falciparum* or* P. vivax* can be used to measure the level of disequilibrium between a known set of polymorphic markers [45–47]. Disequilibrium occurring between two or more putative drug resistance genes can be used to (i) identify the genotypes of emerging MDR parasites and (ii) determine the extent to which the geographically associated or prone MDR haplotypes of* P. falciparum* or* P. vivax* exist in various malaria ecotopes. On the other hand, if* Anopheles* ecotypes within a given species are able to be adapted to specific malaria ecotopes referred to as the MDR malaria transmission foci, the most likely explanation is that there will be a number of subpopulations of geographically prone MDR malaria parasites. The presence of linkage disequilibrium between a set of those polymorphic markers identifies the chromosomal region spanned by the markers as a candidate location of the MDR genes or high-density single nucleotide polymorphic (SNP) genotypes or used to reconstruct the modality of recombination [45–47].

## 4. Molecular Xenomonitoring of MDR Malaria Parasites in* Anopheles *Vectors

### 4.1. EES and Infection Pocket

The GMS and Southeast Asia are likely to be the epicenters of* P. falciparum* and* P. vivax* MDR malaria parasites as the spread of emergent MDR malaria becomes increasingly important [2–8]. As mentioned earlier, the EES and molecular xenomonitoring (i.e., nucleic acid detection and differentiation of malaria parasites present in mosquitoes) of MDR malaria parasites in* Anopheles* vectors [48, 49] can provide the proof of potential transmission and spread of MDR malaria parasites in any given malaria ecotope and ecotone. During a peak of seasonal malaria transmission in endemic localities, an increasing number of malaria suspected cases that seek blood examinations and treatment at any peripheral healthcare facilities can be used to determine the extent to which the abundance and distribution of* Anopheles* vectors exist in the infection pockets. Also, the vigilance for malaria carriers who do not seek or delay diagnosis and treatment needs to be warranted in endemic localities. However, there is no evidence that the malaria case number relates to the proportion of infected* Anopheles* vectors in the infection pockets. That is, a number of* Anopheles* vectors—whether periodically wild caught using mosquito trapping devices, animal baits, or human landing catches—are more likely to be stochastic from one pocket to another than stable. Mosquito trapping devices and animal bait catches using cattle or swine are likely to be used in outdoor collections of both potent* Anopheles* vectors and their counterparts whether or not they behave anthropophagically. Human landing catches are more likely to be used in the indoor and outdoor collections of anthropophilic* Anopheles* vectors (Figure 2(a)); that is, accurate estimates of the* Plasmodium* infection in potent* Anopheles* vectors are measured as the infection and infectivity rates.

In practice, the infection pocket (Figures 1(b) and 1(c)) is specifically determined by covering the catchment area of possible human-vector contact during which treated malaria cases are followed within 14 days or 28 days. The catchment, approximately a radius of 20 to 200 meters from a malaria patient's house, is normally situated with shaded environments close to a breeding site as it may not cover a segment of adjacent or detached houses. Malaria cases—when improperly conducting protective behaviors—are likely to have an increased risk of possible human-vector contact in the presence of improper household-level implementation of vector control strategies and other personally focused preventive measures. A 14-day follow-up of treatment is crucial for the field staff (e.g., researchers, entomologists, and well-trained vector/infection control personnel) to collect a number of potent* Anopheles* vectors because any susceptible ones—those taking any gametocyte-infected human blood meal before or after treatment—may harbor an enormous number of the sporozoites developing into salivary gland from 6 to 12 days [50–52]. In the largely outdoor environments, parous female adults of* Anopheles* vectors—possibly breeding their progeny one or more times—may or may not have fed human blood meal(s) during their fecundic lifespan. Sporozoite-carrying ones are not indicative of anthropophagically induced parity rate. However, the positive one is epidemiologically important for the isolation of* Plasmodium* infection origin whether or not the source carries single-clone or multiple clonal populations [53, 54]. At the time of mosquito collection, 12-hour human landing catch is performed on 2 to 3 consecutive nights if climatic environmental conditions are suitable. If two or more selected pockets are used in comparable catchments for which particular local environments are considered, complete notes on environmental and climatic conditions as well as meteorologically measured parameters should be recorded along with photographic evidence and georeference.

### 4.2. Molecular Marker-Based PCR Detection Methods Using Salivary Gland DNAs of* Anopheles* Vectors

The molecular basis of genetically determined MDR in* P. falciparum* or* P. vivax* has been linked to the mutations of putative drug resistance genes responsible for intraparasitic pumps that can modulate the transport of the antimalarial drugs and for encoded metabolic enzymes that can reduce selectivity of the drugs. Molecular marker-based PCR methods for the detection and identification of drug resistance genes (Table 1) have been proven to be useful in epidemiological surveillance and monitoring of MDR falciparum and vivax malaria parasites present in the patients as well as in the EES and molecular xenomonitoring of these MDR malaria parasites present in the* Anopheles* vectors. In latter sense, the wild-caught pools or individuals of* Anopheles* vectors provide the isolation sources for species identification, salivary gland (SG) isolation, and isolation and purification of SG DNA extract subject to subsequent molecular marker-based PCR methods using SG DNA (Figure 2(a)).

To achieve the ultimate goal of molecular xenomonitoring of MDR malaria parasites in* Anopheles* vectors, the wild-caught pools of* Anopheles* vectors initially knocked out by cool temperature or anesthesia can then be processed using downstream procedures as mentioned earlier (Figure 2(a)). A SG DNA originally obtained from* Anopheles* vector individuals is subjected to be initially amplified using the small subunit ribosomal RNA (*ssrRNA*) gene-based nested PCRs with genus- and species-specific primer sets according to the methods and procedures described by Singh et al. [75]. For instance, a positive SG DNA could yield a relatively large DNA fragment authentically derived from any orthologous* ssrRNA* genes of four human malaria parasites (*P. falciparum*,* P. vivax*,* P. malariae*, and* P. ovale*) in first-round PCR. In 5 separate second-round PCRs containing another* Plasmodium*-specific primer set and 4 other species-specific primer sets, the internal sequences of this primary PCR product are authentically amplified to yield a* Plasmodium*-specific DNA fragment and other species-specific DNA fragment originated from* P. falciparum*,* P. vivax*,* P. malariae*, or* P. ovale*. If it is positive with primers specific for* P. falciparum* or* P. vivax*, SG DNA template is further used in molecular marker-based PCR analysis of the presence of putative drug resistance genes (Table 1).

**Figure 3:**
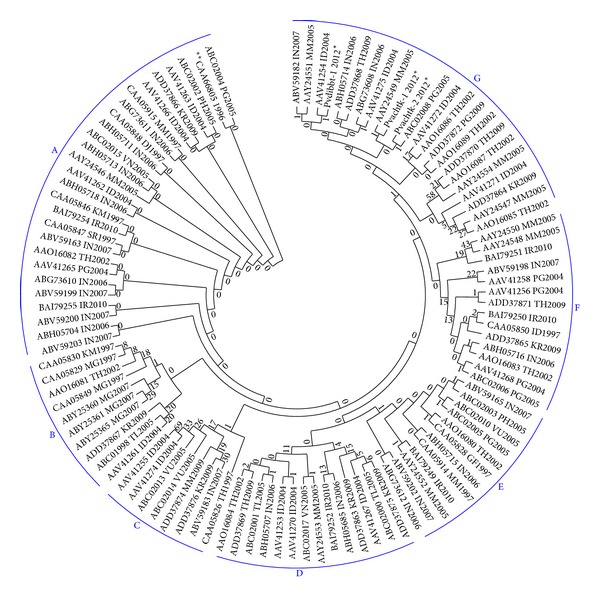
Phylogenetic relationship of* P. vivax* dihydrofolate reductase (DHFR) homolog. The multiple sequence alignment of all representative DHFR homologs of* P. vivax* MDR malaria parasite populations was carried out at both nucleotide and amino acid levels. The phylogenetic reconstruction of haplotypes, which was tested 1000 times with bootstrap method, was constructed based on the maximum parsimony method by using the MEGA ver. 5.22 [76]. DHFR haplotypes and haplogroups (A to G) of geographically prone* P. vivax* parasites conferring point mutations responsible for resistance against antifols and sulfones/sulfonamides are shown in supplementary File S4 (see Supplementary Material available online at http://dx.doi.org/10.1155/2014/969531). As retrieved from the GenBank genome database, all the nucleotide or amino acid sequences (accession numbers) of* P. vivax* DHFR homologs correspond to the infection sources of the geographically prone* P. vivax* isolates including Pvdibbt-1 (KC121333), Pvachtk-1 (KC121334), and Pvachtk-2 (KC121335). The GenBank files of these three isolates are shown in supplementary Files S1 to S3. The qualifier of the submitted sequences pertaining to country or geographic area and submission year was used in the phylogenetic analysis. The isolation sources analyzed include the majority of patient isolates of geographically prone* P. vivax* parasites and a very lesser extent by ∗mosquito isolates and ∗∗laboratory strain of* P. vivax *asexual blood stage. Using standard country codes (http://www.eurogofed.org/calendar/codes.htm), country sources are denoted as DJ: Djibouti, GF: French Guiana, ID: Indonesia, IN: India, IR: Iran, KM: Comoros, KR: South Korea, MG: Madagascar, MM: Myanmar, PG: Papua New Guinea, PH: Philippines, SR: Surinam, SV: El Salvador, TH: Thailand, TL: East Timor, VN: Vietnam, and VU: Vanuatu.

Here, we demonstrate the molecular xenomonitoring of MDR vivax malaria parasite populations in* Anopheles* vectors endogenous to geographically defined ecotopes of malaria-associated rubber plantations in Kanchanaburi province (Figures 1(a)-(a1) and 1(b)), close to Thailand-Myanmar border, as well as in Trat province (Figures 1(a)-(a2) and 1(c)), close to Thailand-Cambodia border. Both malaria endemic provinces experienced increase in malaria incidence among local populations from 2011 to 2012: Kanchanaburi (88 to 132 per 100,000 populations) and Trat (80 to 117 per 100,000 populations). Malaria risk situations are likely due either to malaria transmission dynamic both occurring in certain transmission areas and manifesting a trend of increased incidence, especially* P. vivax*, in transmission-prone areas, or to increased trend of the movement of foreign migrant workers involved in agricultural practices. The entomological data initially recorded in 2009 for these two infection pockets have been described elsewhere [10] and, by using these secondary data, the EES was conducted between September and October 2011. Evidently, the EES established the infection pockets of both malaria ecotopes in which* Anopheles* vectors carrying the* P. vivax* infection can be detected. Within a couple of weeks, during which a course of follow-up treatment was done for the notified* P. vivax* patients that received the first-line treatment,* Anopheles* vectors were collected both indoors and outdoors within a radius of 20 meters from the* P. vivax*-infected patients' houses. The samples were subjected to subsequent identifications as described earlier in Figure 2(a). Both* Plasmodium*-specific nested PCR and molecular marker-based PCR methods—which are based on the genetically polymorphic* Pvdhfr* gene—provide reliable testing results. However, we omitted the likelihood that the* P. vivax* isolate which originated from a positive SG DNA of the sporozoite-carrying* Anopheles* vector may or may not harbor single-clone parasite population. A* Pvdhfr* haplotype (i.e., a set of two or more associated alleles of* Pvdhfr* gene found in the* P. vivax* isolate) is not considered the cost of parasite fitness epidemiologically linked to the human host fitness or the patients infected in a malaria ecotope. Otherwise, the* Pvdhfr* haplotypes found in humans or* Anopheles* vectors in a given malaria ecotope are likely to be the cost of parasite fitness pertaining to the mutations on* Pvdhfr* gene. As seen in Figure 3, the hypothetically potential mutations are involved in amino acid substitutions at codons Phe57Leu/Ile, Ser58Arg, Thr61Met, and Ser117Asn/Thr and, in addition, Pro33Leu, Asp105Asn, Val145Leu, and Ile173Phe/Leu. The other indel (i.e., a segment of gene susceptible to mutation by either insertion or deletion) of a short tandem repeat (NTHGGD) starting from Asn 97 to Asp 102 is considered a neutral allele while the amplification of this short tandem repeat (from 1 to 4 copies) may or may not link to the selection on single, double, triple, or quadruple point mutations (see also Table S4). There is no significant association between the neutral alleles and point mutations whether Phe57Leu/Ile, Ser58Arg, Thr61Met, or Ser117Asn/Thr. As such, the existence of* P. vivax* MDR haplotypes (Figure 3) in the responsible* Anopheles* SG DNAs provides the epidemiological implication that two* Anopheles* vectors had the potential transmission of MDR malaria in the geographically defined ecotopes of malaria-associated rubber plantations on the Thailand-Myanmar border (*An. aconitus*) (Figure 1(b)) as well as the Thailand-Cambodia border (*An. dirus*) (Figure 1(c)).

Moreover, Figure 3 shows a phylogenetic relationship of* P. vivax* DHFR haplotypes geographically associated with the Southeast Asia and with other regions. Six haplogroups (A to G) are thought to be emerging over time as they might evolutionarily escape under the selection in different places and periods. Each haplogroup includes a group of the same or similar haplotype that shares a set of alleles or similar variations. More interestingly,* An. dirus* isolate (Pvdibbt-1) from Thailand-Cambodia border and two* An. aconitus* isolates from Thailand-Myanmar border harbor the haplotype G as in one* An. aconitus* isolate (Pvachtk-1), a newly emerged haplotype conferring two additional point mutations Asp105Asn and Val145Leu which may be the result of the continuation of selective pressures on the associated alleles in malaria ecotope along or close to Thailand-Myanmar border. As noted, such* An. aconitus* can transmit* P. vivax* along the Thailand-Myanmar border although it is known as a secondary vector. As a result of genetic recombination occurring in this vector, this newly emerging* Pvdhfr* haplotype of the hypermutable parasites confers the hexadruple point mutations Leu 57, Arg 58, Met 61, Asn 105, Thr 117, and Leu 145. Like other counterparts, this vector may however transmit the hypermutable* P. vivax* parasites that possess the responsible haplotype through other human blood meals and,* vice versa*, this heritable haplotype may not be selected due to naturally acquired immune defenses in human host fitness (Figure 2(b)).

Taken together, using global alignment of the* Pvdhfr* haplotypes as representative of resistant DHFR homologs of geographically prone* P. vivax* parasites isolated from the GMS and other regions (Figure 3), the phylogenetic analysis of* Pvdhfr* haplotypes can explain (i) the migration or genetic flow of* P. vivax* parasites that is epidemiologically linked to two separate malaria ecotopes and (ii) the magnitude and geographical variation of emergent* P. vivax* haplotypes both within related malaria ecotopes and among diverse malaria ecotopes. Regarding this, the EES and molecular xenomonitoring of MDR vivax malaria parasite support the evidence that there is the association of geographically prone* Pvdhfr* haplotypes of the geographical isolates of* P. vivax* around the globe.

## 5. Perspectives

*P. vivax* malaria parasite becomes increasingly important because its transmission manifests a trend of increasing incidence as well as the spread of chloroquine resistance in* P. vivax* in tropical and subtropical regions of the world [2, 10, 53, 58, 59, 68, 77–82]. As noted earlier, the MDR haplotypes of* P. vivax* that can thrive in the* Anopheles* vectors in a malaria ecotope might be the cost of homozygosity haplotype influenced by the positive selection of pressures including antimalarial drugs, human host immunity, anopheline vector immunity, and phylogenetic constraints over time periods. If the selective pressure increases the fitness of the* P. vivax* population, the mutations occurring in the* P. vivax* MDR haplotypes will result in advantageous MDR genotypes of putative drug resistance genes. Eventually, more* P. vivax* MDR descendants will increase the tolerance of the parasite population and hence decreasing sensitivity to the drugs as multigenic* P. falciparum* MDR parasites do [2]. The intensity of the following selective pressures such as quinolines, antifols, and sulfones/sulfonamides exerts the force of the circumstances such that* P. vivax* MDR parasites will gradually decrease the susceptibility to these drugs* in vitro* and* in vivo*. If there is a balanced selection, the homozygosity of* P. vivax* MDR haplotypes would produce more hypermutable descendants with newly emerging haplotypes, while maintaining the genetic polymorphisms in the population. Without a balanced selection, an appearance of the parasite population bottleneck will eventually reduce the genetic variation in the population. Otherwise the gene pool is much variable in the structure of the parasite subpopulations geographically associated with or prone to transmission foci as* P. falciparum* artemisinin resistance does [74, 83–86].

Together, such development of antifolate resistance in* P. vivax* parasite populations may be the result of a parasite population bottleneck as there is no existence of the wildtype population geographically associated with the locations due to degrees of antifolate pressure. The EES using molecular xenomonitoring tools will help better understand what haplotype is emerging under the selective pressure over time periods in suspected or certain transmission foci, especially on the borders in the GMS where the intensity of antimalarial drug selection pressures and human migration is high. With a prior knowledge of epidemiological and entomological surveillance and monitoring of MDR malaria parasites in malaria ecotopes, we would be able to detect early and monitor in a timely manner MDR malaria parasites geographically associated with the hotspots or prone to the suspected areas in the TCAs or transmission-prone areas of the endemic countries in the phase of malaria control and/or elimination.

## Supplementary Material

Supplement file S1 contains partially coding sequences of a 582−bp DNA fragment with nucleotide substitutions authentically derived from *dhfr* gene of *P. vivax* strain Pvdibbt−1. The sequences of this strain originally isolated from SG DNAs of *An. dirus* were deposited in the genome database under accession no. KC121333.
Supplement files S2-S3 contain partially coding sequences of a 582−bp DNA fragment with nucleotide substitutions authentically derived from *dhfr* gene of *P. vivax* strains Pvachtk−1 and Pvachtk−2. The sequences of these two strains originally isolated from SG DNAs of *An. aconitus* were deposited in the genome database under accession nos. KC121334 and KC121335, respectively.
Supplement file S4 or Table S4 shows that *P. vivax* malaria parasites originally isolated from SG DNAs of An. *dirus* and *An. aconitus* possess *dhfr* homolog with recently emerging haplogroup G as compared to haplogroups A to F.

## Figures and Tables

**Table 1:** Putative drug resistance genes as molecular markers for molecular xenomonitoring of MDR malaria parasites in *Anopheles* vectors.

Class/antimalarial drugs^a^—specific resistance	Annotated drug resistance protein	Annotated orthologous gene	Accession number^g^	Reference
Quinolines and derivatives	Chloroquine resistance transporter^b^	*Pfcrt *	AF030694	[55, 56]
Chloroquine, primaquine, amodiaquine,			AF495378	[57]
and mefloquine		*Pvcrt *	AF314649	[58]
Cinchona alkaloids			EU333972	[59]
Quinine				
Phenanthrenes and derivatives				
Halofantrine, lumefantrine				

Quinolines and derivatives	Multidrug resistance protein^b^	*Pfmdr 1 *	M29154	[60]
Amodiaquine, mefloquine			FJ477805	[61]
Phenanthrenes and derivatives		*Pvmdr 1 *	EU333979	[59]
Lumefantrine	Calcium-dependent	*Pfatp6 *	AB576306	[62]
Sequiterpene lactone	sarcoplasmic/endoplasmic		KC577117	[63]
Artemisinin and derivatives (artesunate,	reticulum ATPase^b^			
artemether)	GTP-cyclohydrolase I^b^	*Pfgch 1 *	AF043557	[64]
Artemisinin-based combination therapies^d^	K13-propeller (Kelch protein)^c^	*PF13_0238 *	AL844509	
(artesunate-amodiaquine, artesunate-			XM001350122	
mefloquine, and artemether-lumefantrine)				

Diazines	Dihydrofolate reductase^e^	*Pfdhfr *	J03028	[65]
Pyrimethamine			J03772	[66]
Benzene and derivatives		*Pvdhfr *	X98123	[67]
Proguanil			DQ514918	[68]

Benzene and derivatives	Dihydropteroate synthase^e^	*Pfdhps *	Z231584	[69]
Sulfadoxine			U07706	[70]
		*Pvdhps *	AY186730	[71]

Acenes and derivatives	Cytochrome *b* ^f^	*Pfcytb *	M9946	[72]
Atovaquone, atovaquone-proguanil		*Pvcytb *	AF055587	[73]

^a^Further details are available at websites: PubChem, http://pubchem.ncbi.nlm.nih.gov/; and DrugBank, http://www.drugbank.ca/.

Molecular mechanism for resistance: ^b^intraparasitic pumps involved in modulation of transporting the drugs across the membranes; ^e^metabolic enzymes involved in decreased selectivity of antifolates and sulfonamides; and ^f^cytochrome *b*c_1_ complex (complex III) involved in decreased selectivity of mitochondrial electron transport inhibitors or ubiquinone analogs.

^c^ *P. falciparum* Kelch protein (encoded by a locus *PF13_0238*) conferring a single point mutation at the position Met476Ile is involved in molecular mechanism for artemisinin resistance [74] as its propensity to the mutation is believed to be the result of the selection under pressures of ^d^ACTs.

^g^Complete genomic DNA sequences served as molecular markers of which design of specific primer sets has been used in monitoring MDR falciparum or vivax malaria parasites present in the patients or *Anopheles* vectors and assessing treatment failure in the patients.
