# Supplementary material for: Ecotope-Based Entomological Surveillance and Molecular Xenomonitoring of Multidrug Resistant Malaria Parasites in Anopheles Vectors
Source: Interdiscip Perspect Infect Dis. 2014 Oct 1;2014:969531. doi: 10.1155/2014/969531 (PMC4198816; doi:10.1155/2014/969531)
Supplement: Supplementary file 1 — Supplement file S1 contains partially coding sequences of a 582−bp DNA fragment with nucleotide substitutions authentically derived from dhfr gene of P. vivax strain Pvdibbt−1. The sequences of this strain originally isolated from SG DNAs of An. dirus were deposited in the genome database under accession no. KC121333. Supplement files S2-S3 contain partially coding sequences of a 582−bp DNA fragment with nucleotide substitutions authentically derived from dhfr gene of P. vivax strains Pvachtk−1 and Pvachtk−2. The sequences of these two strains originally isolated from SG DNAs of An. aconitus were deposited in the genome database under accession nos. KC121334 and KC121335, respectively. Supplement file S4 or Table S4 shows that P. vivax malaria parasites originally isolated from SG DNAs of An. dirus and An. aconitus possess dhfr homolog with recently emerging haplogroup G as compared to haplogroups A to F. [file 969531.f1.pdf]

S1: Plasmodium vivax strain Pvdibbt-1 dihydrofolate reductase (dhfr) gene, partial cds

GenBank: KC121333.1

FASTA Graphics

Go to:

```
LOCUS       KC121333                 582 bp    DNA     linear   INV 06-FEB-2013
DEFINITION  Plasmodium vivax strain Pvdibbt-1 dihydrofolate reductase (dhfr)
            gene, partial cds.
ACCESSION   KC121333
VERSION     KC121333.1  GI:444792510
KEYWORDS    .
SOURCE      Plasmodium vivax (malaria parasite P. vivax)
  ORGANISM  Plasmodium vivax
            Eukaryota; Alveolata; Apicomplexa; Aconoidasida; Haemosporida;
            Plasmodium; Plasmodium (Plasmodium).
REFERENCE   1  (bases 1 to 582)
  AUTHORS   Sorosjinda-Nunthawarasilp,P., Rattaprasert,P.,
            Suntornthiticharoen,P., Markwardt,R.A. and Wihokhern,B.
  TITLE     Direct Submission
  JOURNAL   Submitted (01-NOV-2012) Fundamentals of Public Health, Faculty of
            Public Health, Burapha University, 169 Longhard Bangsean Road,
            Muang, Chonburi 20131, Thailand
COMMENT     ##Assembly-Data-START##
            Sequencing Technology :: Sanger dideoxy sequencing
            ##Assembly-Data-END##
FEATURES             Location/Qualifiers
     source          1..582
                     /organism="Plasmodium vivax"
                     /mol_type="genomic DNA"
                     /strain="Pvdibbt-1"
                     /db_xref="taxon:5855"
                     /PCR_primers="fwd_name: pvh-fw, fwd_seq:
                     atctgcgcatgctgcaag, rev_name: pvh-rv, rev_seq:
                     gaagaagacgtcacacggctag"
     gene            <1..>582
                     /gene="dhfr"
     mRNA            <1..>582
                     /gene="dhfr"
                     /product="dihydrofolate reductase"
     CDS             <1..>582
                     /gene="dhfr"
                     /codon_start=1
                     /product="dihydrofolate reductase"
                     /protein_id="AGE12495.1"
                     /db_xref="GI:444792511"
                     /translation="ICACCKVAPTSEGTKNEPFSPRTFRGLGNKGTLPWKCNSVDMKY
                     IRSVMTYVDESKYEKLKWKRERYLRMEASQGGGDNTSGGDNTHGGDNADKLQNVVVMG
                     RSTWESIPKQYKPLPNRINVVLSKTLTKEDVKEKVFIIDSIDDLLLLLKKLKYYKCFI
                     IGGAQVYRECLSRNLIKQIYFTRINGAYPCDVFF"
     variation       133
                     /gene="dhfr"
                     /replace="t"
     variation       135
                     /gene="dhfr"
                     /replace="c"
     variation       138
                     /gene="dhfr"
                     /replace="c"
     variation       146
                     /gene="dhfr"
                     /replace="c"
     variation       314
                     /gene="dhfr"
```

```
                     /replace="g"
ORIGIN
        1 atctgcgcat gctgcaaggt cgcccccacc agtgaaggga caaagaatga accgttcagc
       61 ccgcggacct ttaggggtct gggcaataag gggactctcc catggaaatg caactccgtc
      121 gatatgaagt acataaggtc ggtgatgacc tacgtggatg agtcaaagta tgagaagcta
      181 aagtggaaga gggagaggta cctacgaatg gaagcctcac aggggggggg tgacaacaca
      241 agcggtggtg acaacacaca cggtggtgac aacgccgaca agctgcaaaa cgtcgtggtc
      301 atggggagaa gcacctggga gagcatcccc aagcagtaca agccgctccc aaacagaatc
      361 aacgtcgtgc tttccaagac gctaacaaag gaagacgtga aggaaaaggt cttcataatt
      421 gacagcatag atgacctact gctgctctta aagaagctga agtactacaa atgcttcatc
      481 attgggggag cacaagttta tagggaatgc ctaagtagaa acttaatcaa gcagatctac
      541 ttcacgagga tcaacggcgc ctacccgtgt gacgtcttct tc
//
```

S2: Plasmodium vivax strain Pvachtk-1 dihydrofolate reductase (dhfr) gene, partial cds

GenBank: KC121334.1

FASTA Graphics

Go to:

```
LOCUS       KC121334                 582 bp    DNA     linear   INV 06-FEB-2013
DEFINITION  Plasmodium vivax strain Pvachtk-1 dihydrofolate reductase (dhfr)
            gene, partial cds.
ACCESSION   KC121334
VERSION     KC121334.1  GI:444792512
KEYWORDS    .
SOURCE      Plasmodium vivax (malaria parasite P. vivax)
  ORGANISM  Plasmodium vivax
            Eukaryota; Alveolata; Apicomplexa; Aconoidasida; Haemosporida;
            Plasmodium; Plasmodium (Plasmodium).
REFERENCE   1  (bases 1 to 582)
  AUTHORS   Sorosjinda-Nunthawarasilp,P., Rattaprasert,P.,
            Suntornthiticharoen,P., Markwardt,R.A. and Wihokhern,B.
  TITLE     Direct Submission
  JOURNAL   Submitted (01-NOV-2012) Fundamentals of Public Health, Faculty of
            Public Health, Burapha University, 169 Longhard Bangsean Road,
            Muang, Chonburi 20131, Thailand
COMMENT     ##Assembly-Data-START##
            Sequencing Technology :: Sanger dideoxy sequencing
            ##Assembly-Data-END##
FEATURES             Location/Qualifiers
     source          1..582
                     /organism="Plasmodium vivax"
                     /mol_type="genomic DNA"
                     /strain="Pvachtk-1"
                     /db_xref="taxon:5855"
                     /PCR_primers="fwd_name: pvh-fw, fwd_seq:
                     atctgcgcatgctgcaag, rev_name: pvh-rv, rev_seq:
                     gaagaagacgtcacacggctag"
     gene            <1..>582
                     /gene="dhfr"
     mRNA            <1..>582
                     /gene="dhfr"
                     /product="dihydrofolate reductase"
     CDS             <1..>582
                     /gene="dhfr"
                     /codon_start=1
                     /product="dihydrofolate reductase"
                     /protein_id="AGE12496.1"
                     /db_xref="GI:444792513"
                     /translation="ICACCKVAPTSEGTKNEPFSPRTFRGLGNKGTLPWKCNSVDMKY
                     IRSVMTYVDESKYEKLKWKRERYLRMEASQGGGDNTSGGDNTHGGDNANKLQNVVVMG
                     RSTWESIPKQYKPLPNRINVVLSKTLTKEDLKEKVFIIDSIDDLLLLLKKLKYYKCFI
                     IGGAQVYRECLSRNLIKQIYFTRINGAYPCDVFF"
     variation       133
                     /gene="dhfr"
                     /replace="t"
     variation       135
                     /gene="dhfr"
                     /replace="c"
     variation       138
                     /gene="dhfr"
                     /replace="c"
     variation       146
                     /gene="dhfr"
                     /replace="c"
     variation       277
                     /gene="dhfr"
```

```
                     /replace="g"
     variation       314
                     /gene="dhfr"
                     /replace="g"
     variation       397
                     /gene="dhfr"
                     /replace="g"
ORIGIN
        1 atctgcgcat gctgcaaggt cgccccccacc agtgaaggga caaagaatga accgttcagc
       61 ccgcggacct ttaggggtct gggcaataag gggactctcc catggaaatg caactccgtc
      121 gatatgaagt acataaggtc ggtgatgacc tacgtggatg agtcaaagta tgagaagcta
      181 aagtggaaga gggagaggta cctacgaatg gaagcctcac aggggggggg tgacaacaca
      241 agcggtggtg acaacacaca cggtggtgac aacgccaaca agctgcaaaa cgtcgtggtc
      301 atggggagaa gcacctggga gagcatcccc aagcagtaca agccgctccc aaacagaatc
      361 aacgtcgtgc tttccaagac gctaacaaag gaagacctga aggaaaaggt cttcataatt
      421 gacagcatag atgacctact gctgctctta aagaagctga agtactacaa atgcttcatc
      481 attgggggag cacaagttta tagggaatgc ctaagtagaa acttaatcaa gcagatctac
      541 ttcacgagga tcaacggcgc ctacccgtgt gacgtcttct tc
//
```

S3: Plasmodium vivax strain Pvachtk-2 dihydrofolate reductase (dhfr) gene, partial cds

GenBank: KC121335.1

FASTA Graphics

Go to:

```
LOCUS       KC121335                 582 bp    DNA     linear   INV 06-FEB-2013
DEFINITION  Plasmodium vivax strain Pvachtk-2 dihydrofolate reductase (dhfr)
            gene, partial cds.
ACCESSION   KC121335
VERSION     KC121335.1  GI:444792514
KEYWORDS    .
SOURCE      Plasmodium vivax (malaria parasite P. vivax)
  ORGANISM  Plasmodium vivax
            Eukaryota; Alveolata; Apicomplexa; Aconoidasida; Haemosporida;
            Plasmodium; Plasmodium (Plasmodium).
REFERENCE   1  (bases 1 to 582)
  AUTHORS   Sorosjinda-Nunthawarasilp,P., Rattaprasert,P.,
            Suntornthiticharoen,P., Markwardt,R.A. and Wihokhern,B.
  TITLE     Direct Submission
  JOURNAL   Submitted (01-NOV-2012) Fundamentals of Public Health, Faculty of
            Public Health, Burapha University, 169 Longhard Bangsean Road,
            Muang, Chonburi 20131, Thailand
COMMENT     ##Assembly-Data-START##
            Sequencing Technology :: Sanger dideoxy sequencing
            ##Assembly-Data-END##
FEATURES             Location/Qualifiers
     source          1..582
                     /organism="Plasmodium vivax"
                     /mol_type="genomic DNA"
                     /strain="Pvachtk-2"
                     /db_xref="taxon:5855"
                     /PCR_primers="fwd_name: pvh-fw, fwd_seq:
                     atctgcgcatgctgcaag, rev_name: pvh-rv, rev_seq:
                     gaagaagacgtcacacggctag"
     gene            <1..>582
                     /gene="dhfr"
     mRNA            <1..>582
                     /gene="dhfr"
                     /product="dihydrofolate reductase"
     CDS             <1..>582
                     /gene="dhfr"
                     /codon_start=1
                     /product="dihydrofolate reductase"
                     /protein_id="AGE12497.1"
                     /db_xref="GI:444792515"
                     /translation="ICACCKVAPTSEGTKNEPFSPRTFRGLGNKGTLPWKCNSVDMKY
                     IRSVMTYVDESKYEKLKWKRERYLRMEASQGGGDNTSGGDNTHGGDNADKLQNVVVMG
                     RSTWESIPKQYKPLPNRINVVLSKTLTKEDVKEKVFIIDSIDDLLLLLKKLKYYKCFI
                     IGGAQVYRECLSRNLIKQIYFTRINGAYPCDVFF"
     variation       133
                     /gene="dhfr"
                     /replace="t"
     variation       135
                     /gene="dhfr"
                     /replace="c"
     variation       138
                     /gene="dhfr"
                     /replace="c"
     variation       146
                     /gene="dhfr"
                     /replace="c"
     variation       314
                     /gene="dhfr"
```

```
                     /replace="g"
ORIGIN
        1 atctgcgcat gctgcaaggt cgcccccacc agtgaaggga caaagaatga accgttcagc
       61 ccgcggacct ttaggggtct gggcaataag gggactctcc catggaaatg caactccgtc
      121 gatatgaagt acataaggtc ggtgatgacc tacgtggatg agtcaaagta tgagaagcta
      181 aagtggaaga gggagaggta cctacgaatg gaagcctcac aggggggggg tgacaacaca
      241 agcggtggtg acaacacaca cggtggtgac aacgccgaca agctgcaaaa cgtcgtggtc
      301 atggggagaa gcacctggga gagcatcccc aagcagtaca agccgctccc aaacagaatc
      361 aacgtcgtgc tttccaagac gctaacaaag gaagacgtga aggaaaaggt cttcataatt
      421 gacagcatag atgacctact gctgctctta aagaagctga agtactacaa atgcttcatc
      481 attgggggag cacaagttta tagggaatgc ctaagtagaa acttaatcaa gcagatctac
      541 ttcacgagga tcaacggcgc ctacccgtgt gacgtcttct tc
//
```

S4: *P. vivax* malaria parasites originally isolated from SG DNAs of *An. dirus* and *An. aconitus* that possesses DHFR homolog with recently emerging haplogroup G as compared to haplogroups A to F

<table>
<tr><th rowspan="2">Haplo-group</th><th rowspan="2">Protein Accession</th><th rowspan="2">Genome Accession</th><th rowspan="2">Source</th><th colspan="9">[a]Mutation of haplotype</th></tr>
<tr><th>33</th><th>57</th><th>58</th><th>61</th><th>ΔIndel</th><th>105</th><th>117</th><th>145</th><th>173</th></tr>
<tr><td>A</td><td>ABC02004</td><td>DQ244111</td><td>PG2005</td><td>P</td><td>F</td><td>S</td><td>T</td><td>NTHGGD</td><td>D</td><td>S</td><td>V</td><td>I</td></tr>
<tr><td></td><td>CAA66805</td><td>X98123</td><td>1996**</td><td>P</td><td>F</td><td>S</td><td>T</td><td>**NTSGGD**|NTHGGD</td><td>D</td><td>S</td><td>V</td><td>I</td></tr>
<tr><td></td><td>ABC02002</td><td>DQ244109</td><td>PH2005</td><td>P</td><td>F</td><td>S</td><td>T</td><td>**NTSGGD**|NTHGGD</td><td>D</td><td>S</td><td>V</td><td>I</td></tr>
<tr><td></td><td>AAV41263</td><td>AY772075</td><td>ID2004</td><td>P</td><td>F</td><td>S</td><td>T</td><td>NT**S**GGD</td><td>D</td><td>S</td><td>V</td><td>I</td></tr>
<tr><td></td><td>ADD37866</td><td>GU224157</td><td>KR2009</td><td>P</td><td>F</td><td>S</td><td>T</td><td>NT**S**GGD</td><td>D</td><td>S</td><td>V</td><td>I</td></tr>
<tr><td></td><td>AAV41266</td><td>AY772078</td><td>ID2004</td><td>P</td><td>F</td><td>S</td><td>T</td><td>**NTSGGD**|NTHGGD</td><td>D</td><td>S</td><td>V</td><td>I</td></tr>
<tr><td></td><td>CAA05915</td><td>AJ003157</td><td>MM1997</td><td>P</td><td>F</td><td>S</td><td>T</td><td>**NTSGGD**|NTHGGD</td><td>D</td><td>S</td><td>V</td><td>I</td></tr>
<tr><td></td><td>ABG73611</td><td>DQ679978</td><td>IN2006</td><td>P</td><td>F</td><td>S</td><td>T</td><td>NTHGGD</td><td>D</td><td>S</td><td>V</td><td>I</td></tr>
<tr><td></td><td>CAA05848</td><td>AJ003075</td><td>DJ1997</td><td>P</td><td>F</td><td>S</td><td>T</td><td>NTHGGD</td><td>D</td><td>S</td><td>V</td><td>I</td></tr>
<tr><td></td><td>ABH05711</td><td>DQ789665</td><td>IN2006</td><td>P</td><td>F</td><td>S</td><td>T</td><td>NTHGGD</td><td>D</td><td>S</td><td>V</td><td>I</td></tr>
<tr><td></td><td>ABC02015</td><td>DQ244122</td><td>VN2005</td><td>P</td><td>F</td><td>S</td><td>T</td><td>**NTSGGD**|NTHGGD</td><td>D</td><td>S</td><td>V</td><td>I</td></tr>
<tr><td></td><td>ABH05713</td><td>DQ789667</td><td>IN2006</td><td>P</td><td>F</td><td>S</td><td>T</td><td>**NTSGGD|NTHGGD**|NTHGGD</td><td>D</td><td>S</td><td>V</td><td>I</td></tr>
<tr><td></td><td>AAY24546</td><td>AY942925</td><td>MM2005</td><td>P</td><td>F</td><td>S</td><td>T</td><td>**NTSGGD**|NTHGGD</td><td>D</td><td>S</td><td>V</td><td>I</td></tr>
<tr><td></td><td>AAV41262</td><td>AY772074</td><td>ID2004</td><td>P</td><td>F</td><td>S</td><td>T</td><td>NTHGGD</td><td>D</td><td>S</td><td>V</td><td>I</td></tr>
<tr><td></td><td>ABH05718</td><td>DQ789672</td><td>IN2006</td><td>P</td><td>F</td><td>S</td><td>T</td><td>**NTSGGD**|NTHGGD</td><td>D</td><td>S</td><td>V</td><td>I</td></tr>
<tr><td></td><td>CAA05846</td><td>AJ003073</td><td>KM1997</td><td>P</td><td>F</td><td>S</td><td>T</td><td>**NTSGGD**|NTHGGD</td><td>D</td><td>S</td><td>V</td><td>I</td></tr>
<tr><td></td><td>BAI79254</td><td>AB547457</td><td>IR2010</td><td>P</td><td>F</td><td>S</td><td>T</td><td>**NTSGGD|NTHGGD**|NTHGGD</td><td>D</td><td>S</td><td>V</td><td>I</td></tr>
<tr><td></td><td>CAA05847</td><td>AJ003074</td><td>SR1997</td><td>P</td><td>F</td><td>S</td><td>T</td><td>**NTSGGD**|NTHGGD</td><td>D</td><td>S</td><td>V</td><td>I</td></tr>
<tr><td></td><td>ABV59163</td><td>EU145907</td><td>IN2007</td><td>P</td><td>F</td><td>S</td><td>T</td><td>NTHGGD</td><td>D</td><td>S</td><td>V</td><td>I</td></tr>
<tr><td></td><td>AAO16082</td><td>AF525814</td><td>TH2002</td><td>P</td><td>F</td><td>S</td><td>T</td><td>**NTSGGD**|NTHGGD</td><td>D</td><td>S</td><td>V</td><td>I</td></tr>
<tr><td></td><td>AAV41265</td><td>AY772077</td><td>PG2004</td><td>P</td><td>F</td><td>S</td><td>T</td><td>**NTSGGD|NTSGGD|**
NTHGGD|DTHGGD</td><td>D</td><td>S</td><td>V</td><td>I</td></tr>
<tr><td></td><td>ABG73610</td><td>DQ679977</td><td>IN2006</td><td>P</td><td>F</td><td>S</td><td>T</td><td>**NTSGGD**|NTHGGD</td><td>D</td><td>S</td><td>V</td><td>I</td></tr>
<tr><td></td><td>ABV59199</td><td>EU145943</td><td>IN2007</td><td>P</td><td>F</td><td>S</td><td>T</td><td>**NTSGGD|NTHGGD**|NTHGGD</td><td>D</td><td>S</td><td>V</td><td>I</td></tr>
<tr><td></td><td>BAI79255</td><td>AB547458</td><td>IR2010</td><td>P</td><td>F</td><td>S</td><td>T</td><td>**NTSGGD**|NTHGGD</td><td>D</td><td>S</td><td>V</td><td>I</td></tr>
<tr><td></td><td>ABV59200</td><td>EU145944</td><td>IN2007</td><td>P</td><td>F</td><td>S</td><td>T</td><td>NT**S**GGD</td><td>D</td><td>S</td><td>V</td><td>I</td></tr>
<tr><td></td><td>ABH05704</td><td>DQ789658</td><td>IN2006</td><td>P</td><td>F</td><td>S</td><td>T</td><td>NT**S**GGD</td><td>D</td><td>S</td><td>V</td><td>I</td></tr>
<tr><td></td><td>ABV59203</td><td>EU145947</td><td>IN2007</td><td>P</td><td>F</td><td>S</td><td>T</td><td>**NTSGGD**|NTHGGD</td><td>D</td><td>S</td><td>V</td><td>I</td></tr>
<tr><td>B</td><td>CAA05830</td><td>AJ003054</td><td>KM1997</td><td>**L**</td><td>F</td><td>S</td><td>T</td><td>**NTSGGD**|NTHGGD</td><td>**N**</td><td>S</td><td>V</td><td>I</td></tr>
<tr><td></td><td>CAA05829</td><td>AJ003053</td><td>MG1997</td><td>**L**</td><td>F</td><td>S</td><td>T</td><td>**NTSGGD**|NTHGGD</td><td>D</td><td>S</td><td>V</td><td>I</td></tr>
<tr><td></td><td>AAO16081</td><td>AF525813</td><td>TH2002</td><td>**L**</td><td>F</td><td>S</td><td>T</td><td>**NTSGGD**|NTHGGD</td><td>D</td><td>S</td><td>V</td><td>I</td></tr>
<tr><td></td><td>CAA05849</td><td>AJ003076</td><td>MG1997</td><td>**L**</td><td>F</td><td>S</td><td>T</td><td>**NTSGGD**|NTHGGD</td><td>D</td><td>S</td><td>V</td><td>I</td></tr>
<tr><td></td><td>ABY25360</td><td>EU168426</td><td>MG2007</td><td>**L**</td><td>F</td><td>S</td><td>T</td><td>**NTSGGD**|NTHGGD</td><td>D</td><td>S</td><td>V</td><td>I</td></tr>
<tr><td></td><td>ABY25361</td><td>EU168427</td><td>MG2007</td><td>P</td><td>F</td><td>S</td><td>T</td><td>**NTSGGD**|NTHGGD</td><td>D</td><td>**N**</td><td>V</td><td>I</td></tr>
<tr><td></td><td>ABY25365</td><td>EU168431</td><td>MG2007</td><td>P</td><td>F</td><td>**R**</td><td>T</td><td>**NTSGGD**|NTHGGD</td><td>D</td><td>**N**</td><td>V</td><td>I</td></tr>
<tr><td></td><td>ADD37867</td><td>GU224158</td><td>KR2009</td><td>P</td><td>**L**</td><td>S</td><td>T</td><td>**NTSGGD**|NTHGGD</td><td>D</td><td>S</td><td>V</td><td>I</td></tr>
</table>

	ABC01998	DQ244105	TL2005	P	F	S	T	**NTSGGD**|NT**S**GGD	D	S	V	I
	AAV41261	AY772073	ID2004	P	F	S	T	**NTSGGD|NTHGGD**|NT**S**GGD	D	S	V	I
C	AAV41255	AY772067	ID2004	P	**L**	S	T	**NTSGGD**|NTHGGD	D	S	V	**F**
	AAV41274	AY772086	ID2004	P	**L**	S	T	**NTSGGD**|NTHGGD	D	**T**	V	**F**
	ABC02013	DQ244120	VU2005	P	**L**	S	T	**NTSGGD**|NTHGGD	D	**T**	V	**F**
	ABC02014	DQ244121	VU2005	P	**L**	**R**	T	**NTSGGD**|NTHGGD	D	**T**	V	**F**
	ADD37874	GU224165	MM2009	P	F	S	T	**NTSGGD**|NTHGGD	D	S	V	**F**
	ADD37876	GU224167	KR2009	P	**L**	S	T	NT**S**GGD	D	S	V	**F**
	ABV59183	EU145927	IN2007	P	**L**	S	T	**NTSGGD**|NTHGGD	D	S	V	**F**
	CAA05826	AJ003050	TH1997	P	**L**	S	T	**NTSGGD**|NTHGGD	D	S	V	I
D	AAO16084	AF525816	TH2002	P	F	**R**	T	NT**S**GGD	D	**N**	V	I
	ADD37869	GU224160	TH2009	P	F	**R**	T	NT**S**GGD	D	**N**	V	I
	ABC02001	DQ244108	TL2005	P	F	**R**	T	**NTSGGD**|NT**S**GGD	D	**N**	V	I
	ABH05707	DQ789661	IN2006	P	F	**R**	T	NT**S**GGD	D	**N**	V	I
	AAV41253	AY772065	ID2004	P	F	**R**	T	NT**S**GGD	D	S	V	I
	AAV41270	AY772082	ID2004	P	F	**R**	T	**NTSGGD**|NT**S**GGD	D	**N**	V	I
	ABC02017	DQ244124	VN2005	P	F	**R**	T	NT**S**GGD	D	**N**	V	I
	AAY24553	AY942932	MM2005	P	F	**R**	T	NT**S**GGD	D	**N**	V	I
	BAI79252	AB547455	IR2010	P	F	S	T	**NTSGGD**|NTHGGD	D	**N**	V	I
	ABH05685	DQ789639	IN2006	P	F	S	T	**NTSGGD**|NTHGGD	D	**N**	V	I
	ADD37863	GU224154	KR2009	P	F	S	T	NT**S**GGD	D	**N**	V	I
	AAV41267	AY772079	ID2004	P	F	S	T	**NTSGGD|NTSGGD**|NTHGGD	D	**N**	V	I
	ABC02000	DQ244107	TL2005	P	F	S	T	**NTSGGD**|NT**S**GGD	D	**N**	V	I
	ADD37875	GU224166	KR2009	P	F	S	T	**NTSGGD**|NT**S**GGD	D	**N**	V	I
E	ABG73612	DQ679979	IN2006	P	F	**R**	T	**NTSGGD**|NTHGGD	D	**N**	V	I
	ABV59202	EU145946	IN2007	P	F	**R**	T	**NTSGGD**|NTHGGD	D	**N**	V	I
	AAY24552	AY942931	MM2005	P	F	**R**	T	**NTSGGD**|NTHGGD	D	**N**	V	I
	BAI79249	AB547452	IR2010	P	F	**R**	T	**NTSGGD**|NTHGGD	D	**N**	V	I
	CAA05914	AJ003156	MM1997	P	F	**R**	T	**NTSGGD**|NTHGGD	D	**N**	V	I
	ABH05715	DQ789669	IN2006	P	F	**R**	T	**NTSGGD**|NTHGGD	D	**N**	V	I
	CAA05828	AJ003052	GF1997	P	F	**R**	T	**NTSGGD**|NTHGGD	D	**N**	V	**L**
	AAO16080	AF525812	TH2002	P	F	**R**	T	**NTSGGD**|NTHGGD	D	**N**	V	I
	ABC02005	DQ244112	PG2005	P	F	**R**	T	**NTSGGD**|NTHGGD	D	**N**	V	I
	ABC02010	DQ244117	VU2005	P	F	**R**	T	**NTSGGD**|NTHGGD	D	**N**	V	I
	ABC02003	DQ244110	PH2005	P	F	**R**	T	**NTSGGD**|NTHGGD	D	**N**	V	I
	ABV59165	EU145909	IN2007	P	F	S	T	**NTSGGD**|NTHGGD	D	**N**	V	I
F	ABC02006	DQ244113	PG2005	P	**L**	S	T	**NTSGGD**|NTHGGD	D	S	V	I
	AAV41268	AY772080	PG2004	P	**L**	**R**	T	**NTSGGD|NTHGGD**|NTHGGD	D	S	V	I
	AAO16083	AF525815	TH2002	P	**L**	**R**	T	**NTSGGD**|NTHGGD	D	S	V	I
	ABH05716	DQ789670	IN2006	P	**L**	**R**	T	**NTSGGD**|NTHGGD	D	S	V	I
	ADD37865	GU224156	KR2009	P	**L**	**R**	T	NT**S**GGD	D	S	V	I
	CAA05850	AJ003077	ID1997	P	**L**	**R**	T	**NTSGGD**|NTHGGD	D	S	V	I
	BAI79250	AB547453	IR2010	P	**L**	**R**	T	**NTSGGD**|NTHGGD	D	S	V	I

	ADD37871	GU224162	TH2009	P	**L**	**R**	T	**NTSGGD**|NTHGGD	D	S	V	I
	AAV41256	AY772068	PG2004	P	**L**	**R**	T	**NTSGGD**|NTHGGD	D	S	V	I
	AAV41258	AY772070	PG2004	P	F	**R**	T	**NTSGGD**|NTHGGD	D	S	V	I
	ABV59198	EU145942	IN2007	P	F	**R**	T	**NTSGGD**|NTHGGD	D	S	V	I
	BAI79251	AB547454	IR2010	P	**L**	**R**	T	**NTSGGD**|NTHGGD	D	**N**	V	I
	AAY24548	AY942927	MM2005	P	**L**	**R**	**M**	NT**S**GGD	D	**N**	V	I
	AAY24550	AY942929	MM2005	P	**L**	**R**	**M**	**NTSGGD**|NTHGGD	D	**N**	V	I
**G**	AAO16085	AF525817	TH2002	P	F	**R**	T	NT**S**GGD	D	**T**	V	I
	AAY24547	AY942926	MM2005	P	F	**R**	T	NT**S**GGD	D	**T**	V	I
	ADD37864	GU224155	KR2009	P	F	S	T	NT**S**GGD	D	**T**	V	I
	AAV41271	AY772083	ID2004	P	F	**R**	**M**	**NTSGGD**|NTHGGD	D	**T**	V	I
	AAY24554	AY942933	MM2005	P	**I**	**R**	**M**	**NTSGGD**|NTHGGD	D	**T**	V	I
	AAO16087	AF525819	TH2002	P	**I**	**R**	**M**	**NTSGGD**|NTHGGD	D	**T**	V	I
	ADD37870	GU224161	TH2009	P	**I**	**R**	**M**	**NTSGGD**|NTHGGD	D	**T**	V	I
	AAO16089	AF525821	TH2002	P	**L**	**R**	**M**	**NTSGGD**|NTHGGD	D	**T**	V	I
	ADD37872	GU224163	PG2009	P	**L**	**R**	**M**	**NTSGGD|NTHGGD**|NTHGGD	D	**T**	V	I
	AAO16086	AF525818	TH2002	P	**L**	**R**	T	NT**S**GGD	D	**T**	V	I
	AAV41272	AY772084	ID2004	P	**L**	**R**	T	**NTSGGD**|NTHGGD	D	**T**	V	I
	ABC02008	DQ244115	PG2005	P	**L**	**R**	**M**	**NTSGGD|NTHGGD**|NTHGGD	D	**T**	V	I
	Pvachtk-2	KC121335	TH2012*	P	**L**	**R**	**M**	**NTSGGD**|NTHGGD	D	**T**	V	I
	Pvachtk-1	KC121334	TH2012*	P	**L**	**R**	**M**	**NTSGGD**|NTHGGD	**N**	**T**	**L**	I
	AAY24549	AY942928	MM2005	P	**L**	**R**	**M**	NT**S**GGD	D	**T**	V	I
	AAV41275	AY772087	ID2004	P	**L**	**R**	**M**	**NTSGGD**|NTHGGD	D	**T**	V	I
	ABG73608	DQ679975	IN2006	P	**L**	**R**	**M**	**NTSGGD**|NTHGGD	D	**T**	V	I
	ADD37868	GU224159	TH2009	P	**L**	**R**	**M**	**NTSGGD**|NTHGGD	D	**T**	V	I
	ABH05714	DQ789668	IN2006	P	**L**	**R**	**M**	**NTSGGD**|NTHGGD	D	**T**	V	I
	Pvdibbt-1	KC121333	TH2012*	P	**L**	**R**	**M**	**NTSGGD**|NTHGGD	D	**T**	V	I
	AAV41254	AY772066	ID2004	P	**L**	**R**	**M**	**NTSGGD**|NTHGGD	D	S	V	I
	AAY24551	AY942930	MM2005	P	**L**	**R**	**M**	**NTSGGD**|NTHGGD	D	**T**	V	I
	ABV59182	EU145926	IN2007	P	**L**	**R**	**M**	**NTSGGD**|NTHGGD	D	**T**	V	I

Based on the phylogenetic analysis of the representative *Pvdhfr* haplotypes shown in **Figure 3**, *P. vivax* DHFR homologs possess the potential point mutations (blue-color highlighted)[a] or amino acid substitutions at positions Phe57Leu/Ile, Ser58Arg, Thr61Met and Ser117Asn/Thr and, additionally, Pro33Leu, Asp105Asn, Val145Leu, and Ile173Phe/Leu.

[a]Indel shows one short tandem repeat of wild type haplotype (NTHGGD) or prone haplotype (NTSGGD)[a] that possesses the substitution at position His99Ser. Additional short tandem repeats[a] are blue-color highlighted.

*3 haplotypes of *P. vivax* originally isolated from the same 2 *An. aconitus* and one *An. dirus* SG DNA isolates were used in the phylogenetic analysis.
